# Displaced midshaft fractures of the clavicle: non-operative treatment versus plate fixation (Sleutel-TRIAL). A multicentre randomised controlled trial

**DOI:** 10.1186/1471-2474-12-196

**Published:** 2011-08-24

**Authors:** Sylvia A Stegeman, Mireille de Jong, Cornelis FM Sier, Pieta Krijnen, Jan W Duijff, Tom PH van Thiel, Piet AR de Rijcke, Nicolaj MR Soesman, Tjebbe Hagenaars, Freek D Boekhoudt, Mark R de Vries, Gert R Roukema, Andras FK Tanka, Jephta van den Bremer, Hub GWM van der Meulen, Maarten WGA Bronkhorst, Bart A van Dijkman, Stephan WAM van Zutphen, Dagmar I Vos, Niels WL Schep, Martin G Eversdijk, Ger DJ van Olden, Johan GH van den Brand, Robert Jan Hillen, Jan Paul M Frölke, Inger B Schipper

**Affiliations:** 1 Department of Surgery-Traumatology, Leiden University Medical Centre, P.O. box 9600, 2300 RC Leiden, the Netherlands; 2 Department of Surgery, Streekziekenhuis Koningin Beatrix, P.O. box 9005, 7100 GG Winterswijk, the Netherlands; 3 Department of Surgery, IJsselland Hospital, P.O. box 690, 2900 AR Capelle a/d IJssel, the Netherlands; 4 Department of Surgery, Vlietland Hospital, P.O. box 215, 3100 AE Schiedam, the Netherlands; 5 Department of Surgery-Traumatology, Erasmus Medical Centre, P.O. box 2040, 3000 CA Rotterdam, the Netherlands; 6 Department of Surgery, Hospital Gelderse Vallei, P.O. box 9025, 6710 HN Ede, the Netherlands; 7 Department of Surgery, Reinier de Graaf Group, P.O. box 5011, 2600 GA Delft, the Netherlands; 8 Department of Surgery, Maasstad Hospital, P.O. box 9100, 3007 AC Rotterdam, the Netherlands; 9 Department of Surgery, Spaarne Hospital, P.O. box 770, 2130 AT Hoofddorp, the Netherlands; 10 Department of Surgery, Rijnland Hospital, P.O. box 4220, 2350 CC Leiderdorp, the Netherlands; 11 Department of Surgery, Haga Hospital, P.O. box 40551, 2504 LN The Hague, the Netherlands; 12 Department of Surgery, Bronovo Hospital, P.O. box 96900, 2509 JH The Hague, the Netherlands; 13 Department of Surgery, Flevo Hospital, P.O. box 3005, 1300 EG, Almere, the Netherlands; 14 Department of Surgery, Tweesteden Hospital, P.O. box 90107, 5000 LA Tilburg, the Netherlands; 15 Department of Surgery, Amphia Hospital, P.O. box 90158, 4800 RK Breda, the Netherlands; 16 Department of Surgery, Academic Medical Centre, P.O. box 22660, 1100 DD Amsterdam, the Netherlands; 17 Department of Surgery, St Jansdal Hospital, P.O. box 138, 3840 AC Harderwijk, the Netherlands; 18 Department of Surgery, Meander Medical Centre, P.O. box 1502, 3800 BM Amersfoort, the Netherlands; 19 Department of Surgery, Medical Centre Alkmaar, P.O. box 501, 1800 AM Alkmaar, the Netherlands; 20 Department of Orthopaedics, Waterland Hospital, P.O. box 250, 1440 AG Purmerend, the Netherlands; 21 Department of Surgery, Radboud University Nijmegen Medical Centre, P.O. box 9101, 6500 HB Nijmegen, the Netherlands

## Abstract

**Background:**

The traditional view that the vast majority of midshaft clavicular fractures heal with good functional outcomes following non-operative treatment may be no longer valid for all midshaft clavicular fractures. Recent studies have presented a relatively high incidence of non-union and identified speciic limitations of the shoulder function in subgroups of patients with these injuries.

**Aim:**

A prospective, multicentre randomised controlled trial (RCT) will be conducted in 21 hospitals in the Netherlands, comparing fracture consolidation and shoulder function after either non-operative treatment with a sling or a plate fixation.

**Methods/design:**

A total of 350 patients will be included, between 18 and 60 years of age, with a dislocated midshaft clavicular fracture. The primary outcome is the incidence of non-union, which will be determined with standardised X-rays (Antero-Posterior and 30 degrees caudocephalad view). Secondary outcome will be the functional outcome, measured using the Constant Score. Strength of the shoulder muscles will be measured with a handheld dynamometer (MicroFET2). Furthermore, the health-related Quality of Life score (ShortForm-36) and the Disabilities of Arm, Shoulder and Hand (DASH) Outcome Measure will be monitored as subjective parameters. Data on complications, bone union, cosmetic aspects and use of painkillers will be collected with follow-up questionnaires. The follow-up time will be two years. All patients will be monitored at regular intervals over the subsequent twelve months (two and six weeks, three months and one year). After two years an interview by telephone and a written survey will be performed to evaluate the two-year functional and mechanical outcomes. All data will be analysed on an intention-to-treat basis, using univariate and multivariate analyses.

**Discussion:**

This trial will provide level-1 evidence for the comparison of consolidation and functional outcome between two standardised treatment options for dislocated midshaft clavicular fractures. The gathered data may support the development of a clinical guideline for treatment of clavicular fractures.

**Trial registration:**

Netherlands National Trial Register NTR2399

## Background

### Epidemiology

Fractures of the clavicle account for 2.6 to 4 percent of all adult fractures and 35 percent of all injuries to the shoulder girdle [1,2]. The annual incidence of clavicular fractures is estimated between 29 and 64 per 100,000. Fractures of the middle third (midshaft) account for 69 to 82 percent of all clavicular fractures, whereas distal fractures represent 21 to 28 percent. Medial-end injuries are less common, approximately 2 to 3 percent of all clavicular fractures [2,3]. The average age of patients sustaining a midshaft clavicular fracture is 33 years, 70 percent of the patients is male [4]. A fall or a direct blow to the shoulder, giving an axial compressive force on the clavicle, is the most common trauma mechanism of injury for any clavicular fracture [5-7].

### Current treatment concepts

Midshaft fractures have traditionally been treated non-operatively, even when substantially displaced [8]. The non-operative treatment strategy was based on early reports suggesting that clavicular non-unions are very rare. Clavicular mal-union, if present, was reported as being of radiographic interest only, without clinical importance [9]. Moreover, surgical treatment of acute midshaft fractures was not favoured due to relatively frequent and serious complications such as infection, non-union, pin migration, broken plates, and necessity of removal of hardware [2]. However, the prevalence of non-union or mal-union in dislocated midshaft clavicular fractures after conservative treatment is higher than previously presumed and fixation methods have evolved. Of all midshaft clavicular fractures, about two-thirds end up having some degree of mal-union [5]. Recent studies reported a non-union rate up to 15 percent and more [10,11,4] and a potential 20 to 25 percent decrease in shoulder function and arm strength [4,12,11-17].

The currently described indications for surgical treatment are open fractures, neurovascular involvement, skin compromise and wide separation of bone fragments with soft tissue interposition. Initial clavicular shortening exceeding 20 mm is upcoming as an indication for operative treatment, because shortening caused by dislocation has been associated with potential shoulder dysfunction [18,12]. An associated floating shoulder, or a scapular neck fracture, are relative indications for operative treatment of the clavicular fracture. Non-union and mal-union are mentioned as a delayed indication for operative treatment. If an operation is considered for displaced midshaft clavicular fractures, the preferred method of fixation is reduction and internal fixation by means of wires, pins, or plates with screws.

Valid and scientific evidence showing primary operative intervention to be superior compared to closed treatment for dislocated fractures, still lacks [19,20]. Surgery is accepted more and more as primary treatment for dislocated midshaft clavicular fractures, mainly because the results of non-operative treatment are interpreted as inferior to operative treatment [9,15,21,22]. Several studies have examined the safety and efficacy of primary open reduction and internal fixation (ORIF) for completely displaced midshaft clavicular fractures and have noted a high union rate with a low complication rate [23,24,11,9]. However, all these studies were retrospective and only one recent study prospectively compared locking plate fixation with non-operative treatment [9]. In this multicentre, prospective randomised trial 132 patients with a displaced midshaft clavicular fracture were allocated to either operative treatment with plate fixation (n = 67) or non-operative treatment (n = 65). The investigators concluded that operative treatment results in improved functional outcome and a lower rate of mal-union and non-union compared with non-operative treatment after one year of follow-up [9]. One of the important limitations of this prospective randomised trial was a selective loss to follow-up, which occurred predominantly in the non-operatively treated group. This may have obscured the true difference in the outcome parameters between the study groups.

A cost-effectiveness analysis [4] has been performed in this multicentre, prospective randomised trial [9], showing that the cost-effectiveness of ORIF of displaced midshaft clavicular fractures is dependent on the duration and magnitude of functional benefit after ORIF, the disutility before union and increased time to union associated with non-operative treatment, and the actual cost of treatment.

### Rationale for the trial

A multicentre randomised clinical trial with sufficient power is needed to provide scientific support for a preferred treatment strategy for dislocated midshaft fractures of the clavicle. The aim of this trial is to compare the results of plate fixation with non-operative management of dislocated midshaft fractures of the clavicle with respect to the incidence of non-union, functional outcome, pain scores, Quality of Life, cosmetic aspects, and complications.

## Methods/Design

### Study design

The Sleutel-TRIAL is designed as a multicentre randomised controlled trial. In total twenty-one academic and non-academic centres in the Netherlands will participate. The study started 15 June, 2010. The trial has been developed to meet the Declaration of Helsinki (59^th ^World Medical Association General Assembly, Seoul, October 2008) and in accordance with the Medical Research Involving Human Subjects Act [25]. It will follow the CONSORT (CONsolidation of Standards of Reporting Trials) guidelines [26-28].

### Recruitment, consent and randomisation

All eligible persons presenting at the Emergency Department (ED) or at the outpatient clinic with a new, dislocated midshaft clavicular fracture are informed about this trial. They receive information and a consent form from the attending physician, the physician assistant or the clinical investigator. After written informed consent has been obtained, the patient is randomised for either operative therapy with a plate fixation or for non-operative therapy. Minimisation randomisation is accomplished via the trial website using TenALEA (Trans European Network for Clinical Trials Services), an online registration and randomisation program. All patients are randomly allocated to one of the two treatment arms in a 1:1 ratio in each participating hospital. For each subsequent participant the allocation depends on the included participants to minimise the imbalance [29].

### Study population

**Figure 1:**
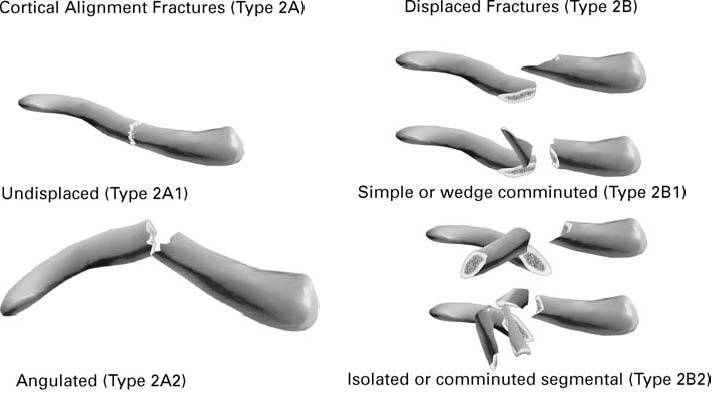
**Robinson Classification type 2 fractures**. Figure reprinted with permission of C.M. Robinson [39]. Right side of the figure shows type 2B1 and type 2B2 fractures.

All patients with a dislocated midshaft clavicular fracture have to meet the following inclusion criteria before enrolment:

1. Fully displaced midshaft fracture (no fracture side contact of distal and proximal fragments) according to Robinson classification 2B1 and 2B2 (see Figure 1). The classification of the fracture will be confirmed on an anterior-posterior X-ray with a 30 degree caudocephalad view;

2. Age between 18 and 60 years;

3. No medical contra-indications to general anaesthesia;

4. Signed informed consent by the patient or a legal representative;

If one of the following exclusion criteria applies, the patient is not eligible for the study:

1. Fracture in the proximal or distal third of the clavicle;

2. Pathologic fracture (bony abnormalities at the side of the fracture) or an open fracture;

3. Neurovascular injury of the shoulder region with objective neurological findings on physical examination;

4. Associated head injury (Glasgow Coma Scale < 12);

5. A significant ipsilateral upper extremity fracture, that would delay the functional recovery of the arm;

6. A midshaft clavicular fracture more than 14 days old at first hospital visit;

7. Inability to comply with follow-up;

8. Prior surgery to the shoulder or pre-existing shoulder complaints with subsequent loss of function;

### Interventions

For patients assigned to operative treatment, the procedure of applying the plate is performed according to standard procedures, including the position of the patient (beach chair position) and anaesthesia (i.e., general anaesthesia or interscalene nerve block or a combination of both). All patients admitted to the hospital for operative intervention receive antibiotic prophylactics (single dose) pre-operatively and after operation thromboprophylaxis is applied during the hospital stay (e.g., unfractionated heparin, Low Molecular Weight Heparin (LMWH), or equivalent). All operations are performed by skilled trauma surgeons, i.e. those who have performed more than five operations with a plate fixation, or by surgical residents under supervision of a skilled trauma surgeon. No restrictions are specified regarding the brand of plate fixation that will be used. Patients assigned to conservative therapy wear a sling for the first two weeks.

All patients, in both treatment arms, are advised to mobilise the shoulder functionally without weight bearing during the first six weeks. The exercise protocol consists of pendulum exercises up to functional movements without weight bearing in the first six weeks after trauma or operation. In the first two weeks pendulum exercises are started and more active exercise is initiated between two and four weeks postoperatively or after trauma. After six weeks, initial strengthening is started.

### Outcome measures

The primary outcome is the incidence of non-union. This is determined objectively on X-rays by an independent radiologist and two surgeons, and subjectively by evaluation of the clavicular and the arm function. The function of the arm is measured with the Constant Score. The Constant score consists of four variables, reflecting both function and pain of the shoulder joint [30,31]. The subjective variables in the Constant Score are pain, activities of daily living and arm positioning. The objective variables are range of motion (ROM) without pain and strength [32]. The arm strength is measured with the MicroFET2 (Micro Force, Evaluating and Testing 2, Hoggan Health Industries Inc, West Jordan, UT, USA), a hand-held dynamometer. This device measures the force a patient can produce against the force of the examiner in Newton (N). All arm movements (i.e., retroflexion, anteflexion, abduction, adduction, endorotation and exorotation) are evaluated six weeks after initial trauma or operation in comparison with the contralateral side and thereafter at each follow-up moment. For all measurements the Make Test is used. The Make Test is characterised by the examiner holding the dynamometer stationary while the subject exerts a maximal force against the dynamometer and the examiner [33]. The results produced with the hand-held dynamometer have been shown to be reproducible, especially when measured by one single examiner at each hospital (intra-rater reliability) [33]. The inter- and intra-rater reliability of hand-held dynamometry varied in the range from good to high [34,35].

Secondary outcomes are clinical function measured with the DASH Outcome Measure, pain scores, cosmetic aspects, quality of life and complications of the allocated treatment. The Disabilities of Arm, Shoulder and Hand (DASH) Outcome Measure is a validated 30-item, self-report questionnaire designed to describe the disability experienced by people with upper-limb disorders and to monitor changes in symptoms and function over time. The DASH Outcome Measure consists of two components: the disability/symptom section (30 items) and the optional high performance Sport/Music module (4 items). The questions involve the degree of difficulty in performing a variety of physical activities because of problems with the arm, shoulder, or hand. The severity of pain, activity-related pain, tingling, weakness and stiffness is investigated, as well as the effect of upper limb problems on social activities, work, sleep, and self-image [36,32,37]. The questionnaire is filled out at each follow-up moment.

Cosmetic aspects are included in the follow-up questionnaires. These questions are subjective and involve satisfaction with the appearance of the shoulder with and without surgery. The Health Related Quality of Life (HR-QOL) will be evaluated using the Short Form-36 (SF-36). The SF-36 is a validated survey on general health with 36 questions, representing eight health domains that are combined into a physical and a mental component scale [38]. The Physical Component Scale (PCS) contains the health domains physical functioning, role limitations due to physical health, bodily pain and general health perceptions. The Mental Component Scale (MCS) contains the health domains vitality, energy, fatigue, social functioning, role limitations due to emotional problems and general mental health. Scores ranging from 0 to 100 points are derived for each domain, with lower scores indicating poorer function. These scores will be converted in a norm-based score and compared with the norm values for the general population of the United States (1998), in which each scale was scored to have the same standardized average (50 points) and the same standard deviation (10 points) [32].

### Follow-up of patients

After inclusion, all patients will be followed for two years in total. Patients will visit the outpatient clinic after two weeks, six weeks, three months and one year. After two years an interview by telephone and written survey will be conducted to evaluate two-year functional and mechanical outcome. In the operative group follow-up starts on the day of surgery. For the non-operative group this is the day of inclusion (see Table 1).

**Table 1:** Flowchart Sleutel-TRIAL

Date									
Visit	1 Emergency Room (ER)	2 Phone call 48 hours after ER visit	3 † First visit (pre- operative care)	4 † Operation	5 2 weeks	6 6 weeks	7 3 months	8 1 year	9 2 years
Eligible? (checking in- and exclusion	•								
criteria)									
Patient information	•								
Obtaining Informed Consent		•	• ^2^		• ^2^				
Randomisation (operative vs. non-		• ^1^							
operative treatment)									
Case Record Form + Randomisation form			•						
Preparing patient for operation			•						
(aneasthesia i.e.) †									
Peroperative Form †				•					
X-rays	• ^3^			•	• ^4^	• ^3^	•	•	
Follow-up Forms					• ^5^	• ^5^	• ^5^	• ^5^	
DASH-score					•	•	•	•	
SF-36 score (Quality of Life)					•	•	•	•	
Constant score (+MicroFET2)						•	•	•	
Telephone interview and written survey									•

• 1: obtaining Informed Consent (verbally) for randomisation and planning of clinic visit

• 2: obtaining definitive written Informed Consent

• 3: X-rays: AP-view and 30 degrees cephalad view

• 4: Panorama view

• 5: Forms for the corresponding visit

†: only for participants allocated to operative treatment

At each hospital visit various intrinsic (patient-related) and injury-related variables are collected. As part of standard care, X-rays are taken at admission and each follow-up moment. The X-rays are performed in anterior-posterior view and 30° caudocephalad view. After two weeks an X-ray of the contralateral shoulder is taken for comparison with the affected shoulder. The DASH outcome measure and SF-36 are filled out by the patient after two weeks, six weeks, three months and one year. The Constant score of both shoulders is determined after six weeks. The Constant score of the affected shoulder is also determined after three months and one year. The functional tests are performed by a single-blinded researcher or other single-blinded qualified personnel. During these tests, the patients have a sticker on the affected shoulder and they are not allowed to tell the examiner which therapy they have undergone. Furthermore, at each visit the researcher collects medical information according to the follow-up list (i.e., complications/adverse events, secondary interventions). Serious adverse events will be reported directly.

### Sample size calculation

Based on a non-union difference of 15 percent in a previous study [9], the sample size of 175 patients per treatment group was calculated with a power (1-β) of 80 percent and a type I error (α) of 5 percent, allowing for 12 percent drop-out. In total 350 patients will be included.

### Statistical analysis

The research data will be reported following the CONsolidated Standards of Reporting Trial (CONSORT) [26-28]. Complication rates and recovery of function of the shoulder will be compared between the two intervention groups using the Chi-squared test. All other endpoints will be compared using co-variate analysis and student's T-test or Mann-Whitney U-test for, respectively, parametric or non-parametric data. Multivariate linear regression analysis will be performed to model the relation between binary outcome variables and treatment, adjusted for covariates. Data will be presented as mean ± SD (Standard Deviation) for parametric data or medians and percentiles (non-parametric data). P-values lower than 0.05 will be considered statistically significant. The data will be analysed using SPSS version 17 or higher (Statistical Package for the Social Sciences Inc, Chicago IL, USA).

### Ethical considerations

The study will be carried out in compliance with the Declaration of Helsinki on ethical principles for medical research involving human subjects [25]. The Medical Ethics Committee Leiden University Medical Centre (LUMC) acts as central ethics committee for this trial (reference number P10.033 and P10.169; NL31044.058.10 and NL33925.058.10). Approval has also been obtained from the local Medical Ethics Committees of all participating centres. The Medical Ethics Committee LUMC has given dispensation from the statutory obligation to provide insurance for subjects participating in medical research (Medical Research (Human Subjects) Compulsory Insurance Decree of 23 June 2003), because the study concerns two standard treatments and does not introduce extra risks.

## Discussion

The best treatment strategy for dislocated midshaft clavicular fractures remains a topic of debate. Currently, the decision for non-operative or operative treatment of dislocated midshaft clavicular fractures is predominantly based upon the personal preferences of the treating surgeon. In a similar way, when operative treatment is favoured, the type of fixation, intramedullary or (locking) plate fixation, is at the discretion of the surgeon. Research has been done to establish a general consensus on how to treat these types of fractures. The Canadian Orthopaedic Study [9] has provided some insight into how the outcomes after locking plate fixation relate to those after conservative treatment. However, this study has the limitation of a considerable loss to follow-up, predominantly in the non-operatively treated group, which makes it impossible to conclude with certainty that plate fixation is preferred over conservative treatment in active adults. ORIF is most cost-effective for patients who are sensitive to mild functional deficits and strongly value a more rapid return to normal function [4]. Considering these statements, a new randomised controlled trial with sufficient power is needed to provide evidence for a definitive, generally acceptable guideline for the treatment of dislocated midshaft clavicular fractures. The results of this study will help to clarify the question whether plate fixation is superior to non-operative treatment in adults, thereby considering incidence of non-union, functional outcome, pain scores, Quality of Life, cosmetic aspects and complications.

## List of abbreviations used

NTR: Netherlands Trial Registry (in Dutch: Nederlands Trial Register); RCT: Randomized Controlled Trial; ORIF: open reduction and internal fixation; CONSORT: CONsolidated Standards of Reporting Trial; ED: Emergency Department; TenALEA: Trans European Network for Clinical Trials Services; LMWH: Low Molecular Weight Heparin; DASH: Disabilities of the Arm, Shoulder and Hand (DASH) Outcome Measure; HR-QOL: Health-Related Quality of Life; SF-36: Short Form-36; ROM: Range of Motion; MicroFET2: Micro Force, Evaluating and Testing 2; PCS: Physical Component Scale; MCS: Mental Component Scale; SD: Standard Deviation; SPSS: Statistical Package for the Social Sciences; LUMC: Leiden University Medical Centre (in Dutch: Leids Universitair Medisch Centrum);

## Competing interests

This project is supported by Fonds NutsOhra, a non-profit health insurance company in the Netherlands. They contributed to the salary of the principle investigator.

## Authors' contributions

SAS, MJ, JWD, CFMS, JPMF and IBS designed the trial. SAS, MDJ and IBS drafted the manuscript. SAS will act as trial principal investigator. Statistical analyses will be performed by SAS, PK and IBS. TPHT, PARR, NMRS, TH, FDB, MRV, GRR, AFKT, JB, HGWMM, MWGAB, BAD, SWAMZ, DIV, NWLS, MGE, GDJO, JGHB, RJH, JPMF and IBS will perform the surgical procedures and will participate in patient inclusion and assessment. All authors have read and approved the final manuscript.

## Pre-publication history

The pre-publication history for this paper can be accessed here:

http://www.biomedcentral.com/1471-2474/12/196/prepub
